# Potential for adaptive evolution at species range margins: contrasting interactions between red coral populations and their environment in a changing ocean

**DOI:** 10.1002/ece3.1324

**Published:** 2015-02-20

**Authors:** Jean-Baptiste Ledoux, Didier Aurelle, Nathaniel Bensoussan, Christian Marschal, Jean-Pierre Féral, Joaquim Garrabou

**Affiliations:** 1 CIMAR/CIIMAR, Centro Interdisciplinar de Investigacção Marinha e Ambiental, Universidade do Porto, Rua dos Bragas 1774050-123, Porto, Portugal; 2 Institut de Ciencies del Mar CSIC, Passeig Maritim de la Barceloneta 37-49Barcelona, Spain; 3 Aix Marseille Universite, CNRS, IRD, Avignon Université, IMBE, UMR 726313397, Marseille, France; 4 IPSO FACTO, SCOPARL, Pôle Océanologie et Limnologie, 37 rue Saint-SebastienF-13006, Marseille, France

**Keywords:** Common garden, *Corallium rubrum*, deep refugia hypothesis, marginal populations, phenotypic buffering, potential for local adaptation, reciprocal transplants

## Abstract

Studying population-by-environment interactions (PEIs) at species range margins offers the opportunity to characterize the responses of populations facing an extreme regime of selection, as expected due to global change. Nevertheless, the importance of these marginal populations as putative reservoirs of adaptive genetic variation has scarcely been considered in conservation biology. This is particularly true in marine ecosystems for which the deep refugia hypothesis proposes that disturbed shallow and marginal populations of a given species can be replenished by mesophotic ones. This hypothesis therefore assumes that identical PEIs exist between populations, neglecting the potential for adaptation at species range margins. Here, we combine reciprocal transplant and common garden experiments with population genetics analyses to decipher the PEIs in the red coral, *Corallium rubrum*. Our analyses reveal partially contrasting PEIs between shallow and mesophotic populations separated by approximately one hundred meters, suggesting that red coral populations may potentially be locally adapted to their environment. Based on the effective population size and connectivity analyses, we posit that genetic drift may be more important than gene flow in the adaptation of the red coral. We further investigate how adaptive divergence could impact population viability in the context of warming and demonstrate differential phenotypic buffering capacities against thermal stress. Our study questions the relevance of the deep refugia hypothesis and highlights the conservation value of marginal populations as a putative reservoir of adaptive genetic polymorphism.

## Introduction

In the human-dominated Earth ecosystem, global change raises the question of the capacities of populations to cope with the increase of selective pressures (Hoffmann and Sgro 2011). Disentangling the impacts of adaptive processes in population-by-environment interactions (PEIs) is therefore one of the main challenges in evolutionary and conservation biology (Allendorf et al. 2010). In this context, local adaptation, adaptive phenotypic plasticity, and particularly, phenotypic buffering have received increasing attention from both theoretical (Bürger and Lynch 1995) and empirical (Antoniazza et al. 2010) perspectives (Reusch and Wood 2007; Reusch 2014). We define local adaptation as the patterns and processes driven by divergent selection leading to specific PEIs, such that locally adapted individuals exhibit higher relative fitness in their habitat than foreign individuals (Kawecki and Ebert 2004). Adaptive phenotypic plasticity is the capacity for an individual's genotype to produce distinct phenotypes, increasing its relative fitness in response to environmental variations (Via et al. 1995; Pigliucci 2005). The particular case of phenotypic buffering relies on the organism's ability to maintain its physiological functions despite extreme stress (Reusch 2014). Although the interactions between local adaptation and adaptive plasticity are still debated, these two processes are not mutually exclusive (Ghalambor et al. 2007; Baythavong and Stanton 2010). Local adaptation to environmental heterogeneity can rely on adaptive plasticity when heterogeneity is predictable in time over the organism's lifespan (e.g., seasonal heterogeneity; Scheiner 1993) or occurs within populations (i.e., at a scale smaller than gene flow; Baythavong 2011). In addition to the central role of selection, adaptive processes are therefore intrinsically linked to other evolutionary forces, such as gene flow and genetic drift (Alleaume-Benharira et al. 2006; Bridle et al. 2010).

In the study of adaptive processes, the importance of populations at species range margins has recently been emphasized (Hampe and Petit 2005; Kawecki 2008). Compared to core populations, marginal populations have generally been considered to be demographically and genetically impaired because of the combination of low-quality habitats, low densities, and geographic isolation (Lawton 1993). However, some studies have challenged this center-periphery hypothesis (Sagarin and Gaines 2002) and proposed that marginal populations can be locally adapted to the atypical ecological characteristics of their environment (e.g., Garner et al. 2004; Orizaola et al. 2010). These two hypotheses have different implications for conservation biologists. The former implies that marginal populations are vulnerable *per se*, as they are maintained via gene flow from core populations (Hoffmann and Blows 1994). On the contrary, the latter suggests that marginal populations offer the opportunity to characterize the responses of populations facing a new regime of selection and can thus serve as a reservoir of adaptive genetic variation (Hampe and Petit 2005; Kawecki 2008).

The controversial status of marginal populations can deeply impact the development of conservation policies. Focusing on coastal ecosystems, the selective pressures linked to global change (e.g., thermal stress) are stronger in the shallow (0–30 m depth) compared to the mesophotic (30–100 m depth) zone, resulting in an intense decline of shallow compared to mesophotic populations within species (Bongaerts et al. 2010). The deep refugia hypothesis (Glynn 1996) proposes that mesophotic populations could act as refugia and as a source of recruits for shallow populations. Accounting for the bathymetric range of various marine species, we rephrase this hypothesis as the question of whether marginal populations can be replenished by more central ones. Thus far, the deep refugia hypothesis has been tested by searching for neutral connectivity between different bathymetric zones (Bongaerts et al. 2010; Costantini et al. 2011; van Oppen et al. 2011; Brazeau et al. 2013), thus assuming identical PEIs between depths. This does not account for the local adaptations observed from marine taxa (Conover et al. 2006; Marshall et al. 2010; Sanford and Kelly 2010) and at the edge of species range (Kawecki 2008). Therefore, characterization of the PEIs at range margins in marine species is a necessary task to enhance our understanding of the consequences of global change on marine biodiversity and to propose relevant conservation measures.

The Mediterranean red coral, *Corallium rubrum*, is an engineer species in the coralligenous community, which is one of the richest but also most threatened Mediterranean communities (Ballesteros 2006). This species is a sessile, aposymbiotic, and long-lived cnidarian that exhibits slow population dynamics (Garrabou and Harmelin 2002) and late sexual maturity (at 10 years of age; Torrents et al. 2005). *Corallium rubrum* displays a fragmented distribution centered on the western Mediterranean basin and dwells in heterogeneous habitats, as illustrated by its bathymetric distribution, ranging from depths of 5 to 800 m (Costantini et al. 2010). Red coral populations are genetically structured at the scale of tens of meters (Ledoux et al. 2010a; Aurelle et al. 2011; Aurelle and Ledoux 2013), in accordance with the restricted effective dispersal of this species (Ledoux et al. 2010b).

The red coral is overharvested for use in jewelry (Bruckner 2009). Recently, the populations of the northwestern Mediterranean dwelling at the upper edge of this species’ bathymetric distribution (from 5 to 30–50 m, depending on the region) have been impacted by large-scale mass mortality events (Garrabou et al. 2009) linked to positive thermal anomalies in the water column (Bensoussan et al. 2010). These mass mortalities events impacted a large number of species. Within each impacted species, populations were differentially affected by the mortalities (Garrabou et al. 2009). In the red coral, significant differences in the rates of tissue necrosis have been observed between populations and between individuals within populations (Garrabou et al. 2009). *In situ* temperature records (Bensoussan et al. 2010) and *in aquaria* experiments (Torrents et al. 2008) have demonstrated the central role of temperature in these events and revealed that impacted populations are close to the limit of their ecological tolerance.

In this study, we focus on red coral populations dwelling at different depths (shallow = 20 m and mesophotic = 40 m) within the upper edge of this species’ bathymetric range. We combine reciprocal transplants and *in situ* common garden experiments, comparisons between estimates of neutral genetic (*F*_ST_; Wright 1943) and phenotypic (*P*_ST_; Leinonen et al. 2006) differentiation between populations and population genetics analyses to: (1) test the potential for local adaptation at fine spatial scales; (2) reveal the relative impacts of evolutionary forces on the observed patterns; and (3) evaluate the mitigating effects of adaptive processes *in situ* in the context of global change.

## Materials and Methods

### Study system

**Figure 1:**
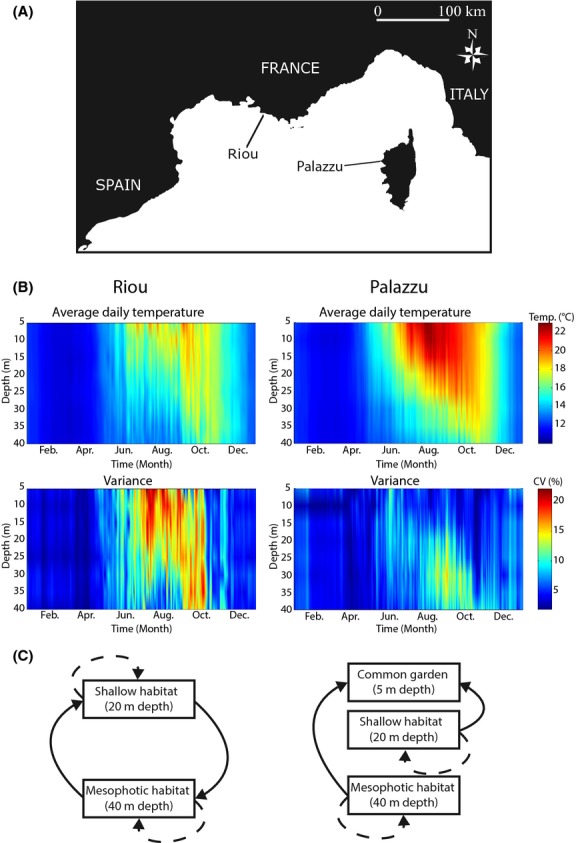
(A) Study sites; (B) Annual thermal regimes: average daily temperature (°C) profiles from 5 to 40 m depths and associated variances (CV in %) computed from hourly times series collected from 1999 to 2012 at Riou and from 2004 to 2011 at Palazzu; (C) Experimental protocols for the RTEs (left panel) and for the CGE (right panel). Dashed and solid arrows represent the control and transplant treatments respectively.

We conducted the study at two localities: Riou Island (RI) (43°10′22.11″N; 5°23′21.93″E) and Palazzu Island (PZ) (42°22′48.61″N; 8°32′46.87″E) (Fig.1A). These localities were chosen because their summer thermal regimes are among the most contrasting in the northwestern Mediterranean (Bensoussan et al. 2010). In each locality and based on a previous population genetics study (Ledoux et al. 2010a), we selected two genetically differentiated red coral populations separated by 100 m, but dwelling at two different depths corresponding to the shallow and mesophotic zones (20 and 40 m designated RI-20, RI-40 and PZ-20, PZ-40). The populations are exposed to different thermal conditions. The shallow habitats (RI-20 and PZ-20) are warmer and more variable than the mesophotic habitats (RI-40 and PZ-40) (Bensoussan et al. 2010; Fig.1B; Table S1a Supplementary information). The shallow populations have been impacted by mass mortality events (Garrabou et al. 2009). They can occasionally be exposed to temperatures above 24°C, which has been proposed as the upper thermal threshold for the red coral (Torrents et al. 2008). In the framework of the deep refugia hypothesis, the mesophotic populations can therefore be considered putative sources of recruits.

### Experimental design

The following experiments were designed to account for the low abundance and low population dynamics of the red coral and the limitations induced by fieldwork conducted via SCUBA diving in the mesophotic habitat (40 m).

#### Reciprocal transplant experiments (RTEs)

We performed two independent RTEs (Fig.1C) at the two localities from June to November 2006, encompassing the summer period. The aim of these experiments was to test the potential for local adaptation of red coral populations. Each RTE included four different treatments: one control (colonies transplanted at their native depth) and one transplant (colonies transplanted at the foreign depth) in each of the two habitats (shallow: 20 m and mesophotic: 40 m). The apical tips of 5 to 7 cm in length (from now on “colony”) of 48 individuals per population were randomly sampled via SCUBA diving and fixed underwater on experimental plates (Appendix S1). Overall, the two RTEs involved 192 colonies from four populations, which were shared among 24 plates, with three plates per treatment and eight colonies per plate.

#### Common garden experiment (CGE)

A CGE (Fig.1C) was conducted on a dimly lit overhang at a depth of five meters to simulate a positive thermal anomaly in situ based on a previous thermal survey (Bensoussan et al. 2010; Fig.1B; Table S1b). The aim of this experiment was to test for differential phenotypic buffering in populations originating from different depths. Because of the logistic constraints linked to the monitoring of the phenotypic response (see below), this CGE was only conducted in Riou. We collected 24 colonies from RI-20 and 24 colonies RI-40 that were randomly distributed among six experimental plates (three per depth of origin). The colonies were individually identified to survey their levels of tissue necrosis and survival from June to September 2006. As a control, we used the control treatments from the RTE.

### Trait measurements

Based on the biological characteristics of the red coral (i.e., low dynamics, longevity, absence of symbiosis) and the depths of the experiments, which limit the fieldwork, we selected three phenotypic traits to assess the impact of environmental conditions on the colonies. We analyzed the mean maximum growth in diameter in the RTEs and the level of tissue necrosis and the survivorship of the colonies in the CGE. These traits are a good proxy for fitness because the reproductive output in gorgonians is function of their size (Hall and Hughes 1996; Linares et al. 2008). Moreover, growth and survival rates are among the most commonly used fitness measures (Leimu and Fischer 2008). Quantification of the mean maximum growth in diameter during the experimental period was made possible by labeling the colonies with calcein prior to transplantation (Appendix S1). At the end of the RTEs, each colony was divided in two parts: The basal portion was air-dried for growth analysis, and the other portion was fixed in 95° ethanol for genotyping (see below). Using stereomicroscope images obtained from sections of the colonies (Appendix S2), we computed the mean maximum growth in diameter as the mean of the 10 greatest distances between the calcein labeling and the periphery of the section. This growth analysis was performed on 71 colonies: Ten per treatment, except for the transplants from 20 m to 40 m, 40 m to 20 m, and the 40 m to 40 m controls at Palazzu in which eight, seven, and six colonies were analyzed due to labeling failure. In the CGE, we surveyed the level of tissue necrosis and the survivorship of the colonies ten times between June and September.

### Microsatellite analysis

Of the 192 individuals utilized in the RTEs, 173 (43, 43, 42, and 45 for RI-20, RI-40, PZ-20, and PZ-40, respectively) were genotyped for 12 microsatellites loci. Due to extensive partial or total mortality of the colonies in the CGE, genotyping was not performed. PCR amplifications were conducted following Ledoux et al. (2010b). To avoid bias due to null alleles, we retained seven loci for statistical analyses (*Mic13*, *Mic20*, *Mic22*, *Mic24*, *Mic26*, *Mic27,* and *COR46bis;* Appendix S3).

### Data analysis

#### Field experiments

In the RTEs, we tested the “local vs. foreign criterion” (Kawecki and Ebert 2004): Local adaptation is supported when local samples show higher fitness than foreign samples in each habitat. The mean maximum growth for each colony in each treatment was considered as the response variable. We considered the data to represent a fully two-way design of samples (replicates) with the “depth of origin” (two levels) and “treatment depth” (two levels) as fixed factors. These data were subjected to a two factorial univariate PERMANOVA test based on Euclidean distance (Anderson 2001; McArdle and Anderson 2001) and accounting for the interaction between both factors. For the CGE, the level of necrosis for each colony was the response variable, and the “depth of origin” was a fixed factor. We conducted a one-way univariate PERMANOVA test based on Euclidean distance (Anderson 2001; McArdle and Anderson 2001). Although the variables were univariate, we conducted PERMANOVA tests because the null distribution of the test statistic in PERMANOVA is produced through permutation (*n *=* *9999) avoiding the normality assumptions required for parametric tests. Analyses were carried out using PRIMER v.6 (Clarke and Gorley 2006) in the PERMANOVA+ module (Anderson et al. 2008).

#### Population genetic analyses

##### Microsatellite characteristics, Hardy–Weinberg equilibrium, genetic diversity, and population structure

The computations conducted for microsatellite characteristics, Hardy–Weinberg equilibrium, and genetic diversity are presented in Appendix S3. Global and pairwise differentiations were computed using the *θ* estimator of *F*_ST_ (Weir and Cockerham 1984) in FREENA (Chapuis and Estoup 2007). We tested for genotypic differentiation in all samples and in all pairs of samples using GENEPOP (Rousset 2008).

To represent the genetic distances between the samples, we produced a phenogram using POPULATIONS 1.2.30 (Langella, 2010) with the neighbor-joining algorithm (Saitou and Nei 1987) and the distance measure of Nei et al. (1983) (*Da*).

##### Impact of migration and drift in ecologically divergent populations

We estimated the level of connectivity for each population using STRUCTURE v.2.3.2 (Pritchard et al. 2000; Falush et al. 2003) by computing the mean percentage of assignment of individuals for each sample. We performed ten runs with a burn-in of 100,000, followed by 300,000 iterations with the number of genetic clusters (*K*) set to 4 (number of populations) using a locprior model (Hubisz et al. 2009), allowing for admixture and correlated allele frequencies between clusters with and without the recessive allele options (Falush et al. 2007). CLUMPP v.1.1 (Jakobsson and Rosenberg 2007) and DISTRUCT v.1.1 (Rosenberg 2004) were employed to average the assignment scores over the ten runs and for graphical display.

The level of genetic drift was estimated using the standard linkage disequilibrium method (Hill 1981) with Waples’ (2006) correction to compute a contemporary value of the effective population size (*N*_*e*_). The computations were performed with LDN_e_ under the random-mating model, excluding rare alleles with frequencies of less than 0.02 and using the jackknife option to estimate confidence intervals (Waples and Do 2008, 2010).

##### *P*_ST_-*F*_ST_ comparisons and sensitivity analysis

Comparison of differentiation measures based on quantitative traits (*Q*_ST_; Spitze 1993) and neutral molecular markers (*F*_ST_) allows the estimation of the relative impacts of neutral and selective processes on populations divergence. Divergent selection is suggested when *Q*_ST_ exceeds *F*_ST_ whereas when *F*_ST_ is greater than *Q*_ST_, stabilizing selection drives the evolution of the considered trait. If the trait evolves neutrally, then *Q*_ST_ should be equal to *F*_ST_ (Leinonen et al. 2013). The computation of *Q*_ST_ requires knowledge on the additive genetic variance within and between populations for the considered trait (Brommer 2011). These components of genetic variance are estimated by performing multigenerational experiments in controlled environments. However, for many species, rearing individuals in the laboratory is not feasible for a variety of reasons such as late sexual maturity, as observed in the red coral (10 years; Torrents et al. 2005). Despite being questioned (see below), the approximation of *Q*_ST_ using *P*_ST_, which measures phenotypic differentiation between populations, has been widely employed for studying the potential for local adaptation in the wild (Leinonen et al. 2008, 2013).

To complement the field experiments, we therefore computed *P*_ST_ values as 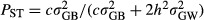, where 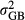 and 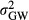 represent the among-sample and the within-sample variance components for the considered phenotypic trait; *h*^2^ represents the assumed additive genetic proportion of differences between individuals within populations (“narrow-sense heritability”); and *c* represents the proportion of the total variance presumed to occur because of additive genetic effects across populations (Brommer 2011). We first considered the null assumption that the genetic architecture of the trait remained equal across populations, with *c/h*^2^ = 1 (Saether et al. 2007; Brommer 2011). For each RTE, we estimated *P*_ST_ between each pair of samples in each habitat using the mean maximum growth in diameter. For the CGE, *P*_ST_ was computed between the two samples based on the level of necrosis within the common garden. The estimators of *F*_ST_ (*θ*) and *P*_ST_ were compared based on their 95% confidence intervals (95% CI). The 95% CI of θ was computed using bootstraps over loci in FREENA. We estimated the 95% CI of the *P*_ST_ by resampling over the maximum growth of the individuals within each sample for the RTEs and over individual necrosis rates within each sample for the CGE.

*P*_ST_*-F*_ST_ comparisons are questioned due to intrinsic characteristics of the two measures (Pujol et al. 2008; Whitlock 2008; Edelaar and Björklund 2011a). *F*_ST_ could be biased for highly polymorphic markers such as microsatellites (Hedrick 2005). Nevertheless, the relevance of alternative measures of population differentiation for multi-allelic markers such as *G*’_ST_ (Hedrick 2005) or *D* (Jost 2008) in *P*_ST_*-F*_ST_ comparisons has been questioned (Leinonen et al. 2008; Edelaar and Björklund 2011a; Edelaar et al. 2011b). Accordingly and following Edelaar and Björklund (2011a), we complemented the comparisons using an allozyme-based *F*_ST_ between populations separated by 80 km (*F*_STall_ = 0.1; Abbiati et al. 1993). Because allozyme loci are less polymorphic than microsatellites and bearing in mind the occurrence of isolation by distance in the red coral (Ledoux et al. 2010a), we considered *F*_STall_ as an upper limit for the genetic differentiation between RI-20 versus RI-40 and PZ-20 versus PZ-40. On the other hand, the approximation conducted using *P*_ST_ instead of *Q*_ST_ can confound the environmental, nonadditive, and additive genetic bases of phenotypic differentiation (Pujol et al. 2008). The accuracy of this approximation is determined by the ratio between *c* and *h*^2^, which are unknown when phenotypic rather than quantitative traits are used (Brommer 2011). Accordingly, Saether et al. (2007) and Brommer (2011) recommended testing the robustness of *P*_ST_*-F*_ST_ comparisons with respect to the variation in *c* and *h*^2^ in the conservative range of values where *c *≤ *h*^2^ (0 < *c/h*^2^ ≤ 1). Considering that the *P*_ST_*-F*_ST_ comparisons were conducted based on the results obtained for populations in the same habitat, the c value is likely to be low. We implemented this sensitivity analysis for the *P*_ST_*-F*_ST_ comparisons that were significant under the null hypothesis (see Results). We evaluated the robustness of each comparison by estimating the 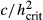 (i.e., the *c/h*^2^ value beyond which *P*_ST_ is significantly higher than *F*_ST_; Brommer 2011) considering the upper limit of the 95% CI of *θ* and *F*_STall_. Considering the intrinsic characteristics of the *P*_ST_ and the *F*_ST_ previously exposed, caution is necessary when interpreting *P*_ST_*-F*_ST_ comparisons. In this context, Brommer (2011) showed that the lower the 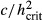 value, the more robust the conclusion regarding the impact of selection on the considered trait and suggested that 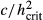 should be lower than 0.2 to draw realistic inferences.

The computations were performed in R (http://cran.r-project.org/), and an example of the scripts used for the *P*_ST_*-F*_ST_ comparisons and the sensitivity analyses is presented in Appendix S4.

## Results

### Reciprocal transplant experiments (RTEs)

**Figure 2:**
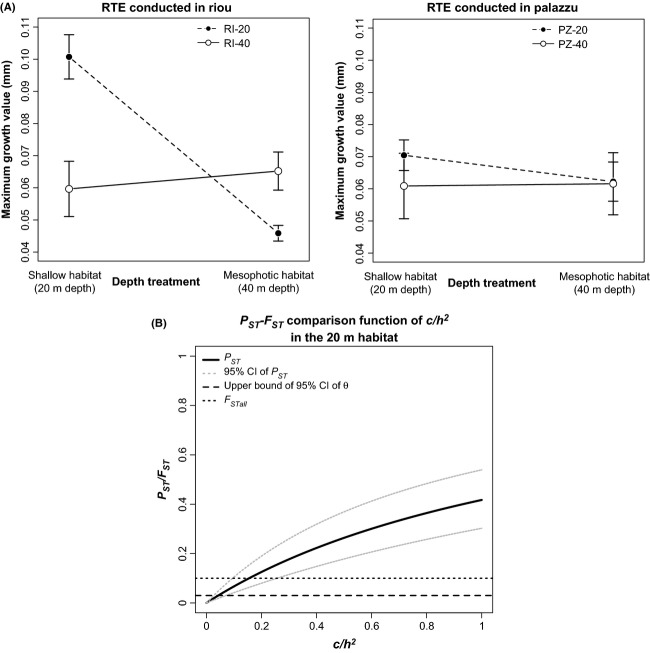
(A) Results of the RTEs based on the mean maximum growth in diameter (bars: standard error; SE = SD/√n with SD = standard deviation and *n* = sample number); (B) Sensitivity analysis of the *P*_ST_-*F*_ST_ comparison conducted for the shallow habitat of the RTE in Riou. The occurrence of divergent selection on the considered trait (growth) is suggested when *P*_ST_ is significantly higher than *F*_ST_. The robustness of the *P*_ST_-*F*_ST_ comparison is tested by considering *c*/*h*^2^ ≤ 1 and by estimating the 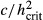 (i.e., the *c/h*^2^ value beyond which *P*_ST_ is significantly higher than *F*_ST_; Brommer 2011) accounting for the upper limit of the 95% CI of *θ* and *F*_STall_.

All the colonies used in the RTEs survived without showing necrosis. At Riou, significant differences in mean maximum growth were observed considering the depth of transplantation (*P *<* *0.01), but not the origin of the sample (*P *=* *0.09). A significant interaction between these two factors (*P* < 0.01) was highlighted. In the two habitats, the local sample exhibited a significantly higher value than the foreign one (Table1a; Fig.2A) supporting the “local vs. foreign criterion”.

**Table 1:** (a) Results of the permutational univariate analyses conducted for the RTEs; (b) results of the PERMANOVA analysis conducted for the CGE.

(a)						
Source of variation	Riou			Palazzu		
	Degrees of freedom	Mean square	Pseudo-F	Degrees of freedom	Mean square	Pseudo-F
Origin	1	1.2 × 10^−3^	2.9 NS	1	1.9 × 10^−4^	0.5 NS
Depth	1	9.1 × 10^−3^	22.5**	1	1.5 × 10^−4^	0.5 NS
Origin × Depth	1	6.1 × 10^−3^	15**	1	1.01 × 10^−4^	0.6 NS
Residual	36	4.1 × 10^−4^		27	4.1 × 10^−4^	
Total	39			30		

(b)			
Source of variation	Degrees of freedom	Mean square	Pseudo-F
Origin	1	37,501	25.10**
Residual	44	1493.3	
Total	45	6.1 × 10^−3^	

NS: nonsignificant

** *P *<* *0.01.

At Palazzu, the growth values were not significantly different according to the origin of the sample (*P *=* *0.49), the transplantation depth (*P *=* *0.55) or their interaction (*P *=* *0.62). Therefore, we could not rejected the null hypothesis of no “local vs. foreign” differences (Table1a; Fig.2A).

### Common garden experiment (CGE)

**Figure 3:**
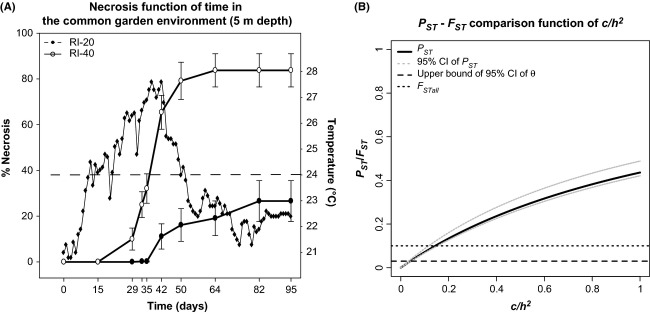
(A) Results of the CGE based on the percentage of necrosis (bars: standard error; SE = SD/√n with SD = standard deviation). Filled (•) and open (○) circles indicate necrosis for RI-20 (*n* = 24) and RI-40 (*n* = 24), respectively: black diamonds (♦) and dashed lines correspond to the temperature measured in the common garden environment and to the thermal threshold of 24°C; (B) Sensitivity analysis of the *P*_ST_-*F*_ST_ comparison. The occurrence of divergent selection on the considered trait (necrosis) is suggested when *P*_ST_ is significantly higher than *F*_ST_. The robustness of the *P*_ST_-*F*_ST_ comparison is tested by considering *c*/*h*^2^ ≤ 1 and by estimating the 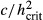 (i.e., the *c/h*^2^ value beyond which *P*_ST_ is significantly higher than *F*_ST_; Brommer 2011) accounting for the upper limit of the 95% CI of *θ* and *F*_STall_.

The colonies in the control treatments did not show any necrosis. The colonies coming from RI-40 showed earlier necrosis than those from RI-20, with the first signs of necrosis being concordant with an increase in temperature beyond 24°C (Fig.3A). At the end of the experiment, the RI-40 colonies were significantly more affected than those from RI-20 (*P *<* *0.01) (Table1b). Moreover, 83.3% (among which 66.7% did not show any necrosis) and 16.6% of the colonies from RI-20 and RI-40, respectively, survived in the common garden.

### Population genetic analyses

No significant linkage disequilibrium was detected among the seven loci when all samples were considered (all *P *>* *0.05 after FDR correction). Significant linkage disequilibrium was observed in PZ-40 for one pair of loci (*Mic22-Mic26*) after FDR correction. The mean (± standard deviation) observed heterozygosity over the loci (*H*_*o*_ Nei 1973) varied from 0.61 ± 0.23 for PZ-40 to 0.66 ± 0.22 for PZ-20, whereas the expected heterozygosity (*H*_e_ Nei 1973) varied from 0.67 ± 0.21 for PZ-20 to 0.76 ± 0.17 for RI-40. Significant heterozygote deficiencies were observed in all samples except PZ-20, with the obtained *f* values ranging from 0.02 for PZ-20 to 0.16 for RI-20 and RI-40. The allelic richness (*Ar*_(64)_) ranged from 9.4 ± 7.6 for PZ-20 to 12.4 ± 9.2 for RI-40, showing a mean value of 10.7 ± 1.3 over the four samples (Table2).

**Table 2:** Genetic characterization of the samples used for the RTEs.

	Genetic diversity				
	Observed heterozygosity *H*_*o*_ (SD)	Gene diversity *H*_*e*_ (SD)	Estimator of *F*_*IS*_ *f*	Allelic richness *Ar*_(64)_ (SD)	Effective population size *N*_*e*_ (95% CI)
Sample name (number of individuals)					
RI-20 (43)	0.63 (0.30)	0.75 (0.16)	0.16***	11 (7.5)	−595.1.7 (226.4–∞)
RI-40 (43)	0.63 (0.28)	0.76 (0.17)	0.16***	12.4 (9.2)	−533.6 (307.5–∞)
PZ-20 (42)	0.66 (0.22)	0.67 (0.21)	0.02 (NS)	9.4 (7.6)	2084.6 (125.6–∞)
PZ-40 (45)	0.61 (0.23)	0.71 (0.2)	0.15***	10 (6.6)	70.8 (45.4–136.2)

NS: nonsignificant deviation from panmixia

*** significant deviation from panmixia at 0.01; SD: standard deviation; 95% CI: 95% confidence interval.

**Figure 4:**
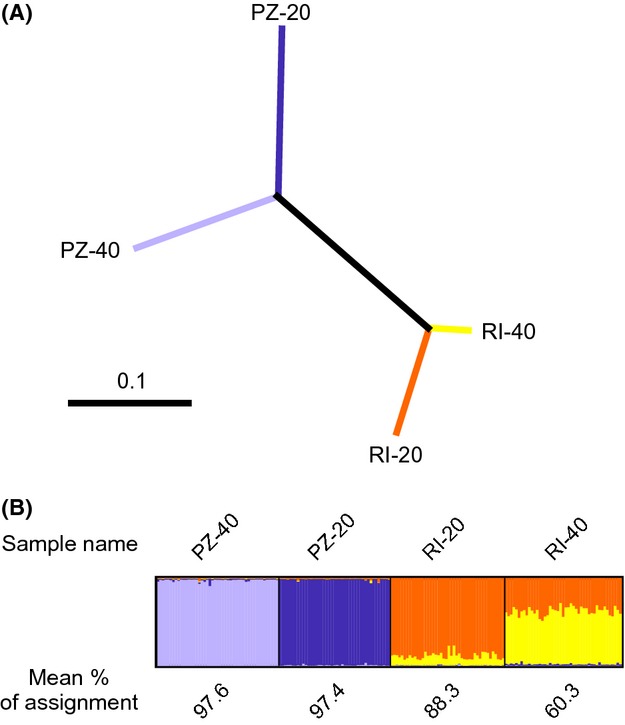
(A) Neighbor-joining phenogram using the distance of Nei et al. (1983) (*Da*). The bootstrap values (*n* = 1000) were equal to 1000 (not shown); (B) Results of the clustering analysis conducted in STRUCTURE for *K* = 4 considering the individuals used in the RTEs. Each individual is represented by a vertical line partitioned into four colored segments that represent the fraction of individual membership in each of the four clusters. Mean percentage of assignment of individuals for each of the four samples is shown above the plot.

The global *θ* was 0.1 (95% CI: 0.07–0.13). The pairwise values and 95% CI for *θ* ranged from 0.02 (95% CI: 0.01–0.03) for RI-20 vs. RI-40 to 0.14 (95% CI: 0.09–0.19) for RI-20 vs. PZ-20. The exact tests for genotypic differentiation were significant at the global level and for the six pairwise comparisons (Table S2). PZ-20 and PZ-40 were the most divergent samples as illustrated by the phenogram (Fig.4A).

The mean percentages of the assignment of the individuals to their sampling localities computed with STRUCTURE were 88.3, 60.3, 97.4, and 97.6% for RI-20, RI-40, PZ-20, and PZ-40, respectively (Fig.4B). The values obtained without the null alleles option were equivalent (not shown). The harmonic mean estimates of *N*_*e*_ ranged from 70.8 for PZ-40 to 2084.6 for PZ-20, with a negative value being obtained RI-20 and RI-40, as expected when the population is sufficiently large that no notable linkage disequilibrium is induced through genetic drift (Waples and Do 2010). The confidence intervals showed an upper limit of infinity, expect for PZ-40. The confidence interval for PZ-40 overlapped with that for PZ-20 but was significantly lower than those for RI-20 and RI-40 (Table2).

### *P*_ST_-*F*_ST_ comparisons and sensitivity analysis

Regarding the RTE conducted at Riou and considering the shallow habitat (20 m) and *c/h*^2^* *=* *1, the *P*_ST_ was 0.42 (95% CI: 0.32–0.53). This *P*_ST_ was significantly higher than *θ* and *F*_STall_ with 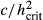 values that were equal to 0.07 and 0.24, respectively (Fig.2B). In the mesophotic habitat (40 m), the *P*_ST_ for *c/h*^2^* *=* *1 was 0.06 (95% CI: 0.01–0.24), which was not significantly different from *θ* and *F*_STall_. Regarding the RTE in Palazzu and considering the shallow habitat and *c/h*^2^* *=* *1, the *P*_ST_ was 0.29 (95% CI: 0.01–0.68), which was not significantly different from *θ* and *F*_STall_. Regarding the RTE in Palazzu, the *P*_ST_ values computed in the shallow and mesophotic habitat were 0. For the CGE, the *P*_ST_ under the null hypothesis was 0.44 (95% CI: 0.42–0.49), which was significantly higher than *θ* and *F*_STall_. The 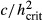 was 0.04 for *θ* and shifted to 0.15 when considering *F*_STall_ (Fig.3B).

## Discussion

Patterns and processes linked to ecology and evolution in marginal habitats are central in our understanding of the responses of natural populations facing global change (Hampe and Petit 2005; Kawecki 2008; Sexton et al. 2009). While neutral processes at species range margin are receiving increasing attention (Eckert et al. 2008), empirical studies examining adaption in these populations remain scarce (Bridle and Vines 2006 but see Kawecki 2008) despite their relevance for the conservation of biodiversity. The present study is a first step toward a better characterization of the processes underlying the interactions between marginal red coral populations and their environment in the context of climate change.

### Contrasting population-by-environment interactions: the potential for local adaptation in marginal red coral populations

Using the mean maximum growth in diameter as a proxy for fitness, we validate the “local vs. foreign criterion” for the RTE conducted at Riou between one shallow and one mesophotic population separated by approximately one hundred meters. This criterion was rejected at Palazzu. Focusing on Riou, two main hypotheses may explain the observed pattern of PEIs. One or both populations may be locally adapted to the environment due to the impact of divergent selection. Alternatively, by chance, other evolutionary forces may induce a pattern of differentiation concordant with the “local vs. foreign criterion” (Kawecki and Ebert 2004). The *P*_ST_-*F*_ST_ comparisons computed for each habitat in the two RTEs allow us to refine this result. Indeed, while no difference was observed at Palazzu, the *P*_ST_ value computed under the null hypothesis (*c/h*^2^* *=* *1) was significantly higher than the *F*_ST_ in the shallow habitat in Riou. The sensitivity analyses showed that this difference between the two measures of differentiation might be considered as relatively robust. Indeed, the 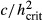 for the shallow habitat when considering *θ* or *F*_STall_ was lower than or slightly superior to 0.2, which is a threshold that has been suggested for realistic approximations of *Q*_ST_ by *P*_ST_ (Brommer 2011). The combination of the RTEs and the *P*_ST_-*F*_ST_ comparisons suggests therefore that some marginal shallow populations of *Corallium rubrum*, such as the RI-20 population, may potentially be locally adapted to their environment. This result fits with the re-evaluation of the importance of local adaptation in the marine realm (Conover et al. 2006; Marshall et al. 2010; Sanford and Kelly 2010). The present study is one of the few to focus on subtidal and benthic species (but see Prada et al. 2008; Bongaerts et al. 2011; Howells et al. 2012; Mariani et al. 2012) and on populations separated by approximately one hundred meters. Nevertheless, considering the limitations linked to the RTE and the *P*_ST_-*F*_ST_ comparisons, complementary studies involving replication at the population level (see Kawecki and Ebert 2004) or genomic approach (Palumbi et al. 2014) are needed to confirm the impact of divergent selection on the observed PEI.

Recent studies demonstrated the occurrence of complex spatial structure of PEIs in various organisms over their whole distribution area or at their range margins (e.g., Willi et al. 2007; Rogell et al. 2010; Kelly et al. 2011; Hice et al. 2012; Vergeer and Kunin 2013). The contrasting patterns of PEIs reported here are concordant with these studies. We can put forth environmentally or genetically based hypotheses to explain this result. The possibility that a lack of divergent selection induced the absence of local adaptation at Palazzu seems unlikely. Indeed, the two habitats show different abiotic properties, as illustrated by the two thermal regimes at PZ-20 and PZ-40, which were more divergent than between RI-20 and RI-40 (Bensoussan et al. 2010). It should therefore be considered that other selective pressures are at play, or, that the populations exhibit different genetic characteristics. Several theoretical studies have demonstrated the importance of the interaction between drift and gene flow in local adaptation (Alleaume-Benharira et al. 2006; Bridle et al. 2010). A meta-analysis of local adaptation also linked the size of populations with their propensity to be locally adapted (Leimu and Fischer 2008). Interestingly, our results support a stronger impact of drift at Palazzu compared to Riou, particularly in PZ-20. PZ-20 and PZ-40 showed the lowest expected heterozygosity and allelic richness and were characterized by the two lowest effective population sizes, and the confidence interval for PZ-40 was significantly smaller than those for RI-20 and RI-40. Moreover, the Palazzu populations were more isolated than those from Riou as illustrated by the higher mean assignment of individuals and genetic divergence. It is also noteworthy that the PZ-20, PZ-40 and RI-20, RI-40 populations belong to two genetic clusters, with the cluster encompassing PZ-20 and PZ-40 being more impacted by drift (Ledoux et al. 2010a). In accordance with model predictions demonstrating that the influence of the effective population size on adaptation increases in poorly connected populations (Alleaume-Benharira et al. 2006; Bridle et al. 2010), we hypothesize that the lack of local adaptation in Palazzu may be related to the stronger influence of the genetic drift on these populations. Accordingly, we posit that adaptive processes of the red coral may be more influenced by genetic drift than by gene flow. If confirmed, this result may have important consequences for the conservation of the species (see below).

### Phenotypic buffering for thermal stress, likely driven by a heterogeneous environment

The *in situ* CGE conducted at Riou supported the occurrence of differential responses to environmental disturbance. The percentage of survival in the stressful environment of the common garden was significantly higher for the shallow colonies than for the mesophotic colonies. The temperature of the common garden was likely a predominant agent in the observed responses. Indeed, the first signs of necrosis in RI-40 coincided with a temperature increase beyond 24°C, which was suggested as a thermal threshold for the red coral (Torrents et al. 2008). Such differential phenotypic buffering between populations facing thermal stress has been previously reported in corals (e.g., Howells et al. 2012) and Mediterranean gorgonians (e.g., Torrents et al. 2008). Focusing on two red coral populations separated by 5 km, Torrents et al. (2008) demonstrated *in aquaria* that the population dwelling in the warmer habitat (10 m) was significantly less affected by a thermal stress than the population dwelling at mesophotic depth (40 m). Our study substantially refines these results because the differential response was observed *in situ* and between individuals originating from shallow and mesophotic populations separated by approximately one hundred meters. This is concordant with the independent functioning of populations suggested by the significant genetic structure reported at this spatial scale (Ledoux et al. 2010a). This is also supported by field surveys conducted after mass mortality events that found contrasting levels of necrosis between populations separated by the same range of distances (Garrabou et al. 2009). Differential phenotypic buffering between populations may be due to distinct processes. In the present case, whether the observed differential phenotypic buffering relies on genetic adaptation or environmental effects remains an open question that requires further studies. Nevertheless, a bunch of evidences allowed us to suggest that a genetic adaptation of the shallow colonies to their local environment may explain their ability to buffer the thermal stress induced by the common garden habitat. First, the difference between the two indices of differentiation, the *F*_ST_ and the *P*_ST_, was significant and robust (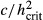 = 0.04), as expected when divergent selection leading to genetic adaptation is involved in the observed pattern (Saether et al. 2007; Brommer 2011; Leinonen et al. 2013). Then, the degree and predictability of environmental heterogeneity seems to be an important driver in the evolution of adaptive plasticity (Scheiner 1993; Via et al. 1995). Interestingly, the thermal regime in the shallow environment is characterized by strong seasonality with more contrasting winter and summer periods than in the mesophotic environment (Bensoussan et al. 2010). Temperature fluctuations associated with nonlethal stress observed in summer in the shallow habitat (Bensoussan et al. 2010) could have beneficial impacts on the red coral colonies, increasing their thermotolerance. This is supported by a recent study focused on the expression of heat shock proteins in the red coral that showed differential expression of HSP70 as a function of the thermal history of individuals (Haguenauer et al. 2013). Beneficial impacts of nonlethal stress on thermotolerance have also been reported for many terrestrial (e.g., Deutsch et al. 2008) and marine species (e.g., Barshis et al. 2010; Oliver and Palumbi 2011; Carilli et al. 2012). Accordingly, we posit that the phenotypic buffering capacities of the shallow colonies might constitute a genetically based adaptation to the seasonal thermal fluctuations observed in the shallow environment. Nevertheless, complementary studies are needed to formally confirm this hypothesis and to test the importance of environmental effects such as maternal effects or epigenetic inheritance on the differential phenotypic buffering.

### Red coral conservation and the deep refugia hypothesis

Complementary studies involving more populations and phenotypic traits are needed to generalize our conclusions. Considering the limitations of the *P*_ST_-*F*_ST_ comparisons, genomic approaches should also be considered to confirm the role played by divergent selection in the observed patterns. Nevertheless, we demonstrated the occurrence of contrasting PEIs with potential for local adaptation at fine spatial scales in a survey involving only four populations. This implies the existence of putatively important adaptive diversity in marginal red coral populations.

From a conservation perspective, genetic drift is an important evolutionary force due to its impact on genetic diversity and inbreeding and, ultimately, on the evolutionary potential of a population (Frankham et al. 2002; Allendorf and Luikart 2007). In this context, our results are particularly relevant. Indeed, genetic drift seems to play a central role in the PEIs in the red coral because the drifting populations of Palazzu are not locally adapted. Conservation priority should thus focus on this evolutionary force by restraining the density erosion of marginal populations directly caused by anthropogenic activities (e.g., harvesting) in order to preserve their evolutionary potential. Additionally, we showed that under realistic environmental stresses, the performance of mesophotic colonies was significantly lower than that of shallow ones. Combining this result with the limited connectivity observed between populations (Ledoux et al. 2010a), the ability of mesophotic populations to replenish shallow populations appears restricted. Using mesophotic populations as a source for restoration efforts aimed at shallow habitats is also expected to be inefficient. Furthermore, we demonstrated the capacity of the shallow population at Riou to cope with the expected increase in sea temperature over short to intermediate timescale (Somot et al. 2008). Shallow populations adapted to marginal environmental conditions could therefore act as reservoir of adaptive genetic variation (Willi et al. 2007; Gienapp et al. 2008). These results unambiguously question the validity of the deep refugia hypothesis for the red coral and other sessile organisms with similar life history traits. Accordingly, we call for the development of studies focused on the adaptive potential of shallow and marginal marine populations to test whether the shallow reservoir hypothesis, rather than the deep refugia hypothesis, should be considered in marine conservation efforts.

## Supporting Information

**Appendix S1.** Experimental plates and calcein labeling.

**Appendix S2.** Analysis of the growth in diameter.

**Appendix S3.** Null allele frequencies, microsatellite characteristics, Hardy-Weinberg equilibrium and genetic diversity.

**Appendix S4.** R-scripts for the *P*_ST_-*F*_ST_ comparisons and corresponding sensitivity analyses.

**Table S1.** (a) Mean temperature conditions from July 1st to September 30th: mean, coefficient of variation (CV) and percentage of time with temperature higher or equal to 20 and 24 °C at 5, 20 and 40 m depth at Riou and 20 and 40 m depth at Palazzu. (b) Mean and maximum temperature values and percentage of time with temperature higher or equal to 20 and 24°C at 5 m depth for the 2006 summer at Riou.

**Table S2.** Pairwise θ values and corresponding 95% CI interval are shown below the diagonal.
