# Supplementary material for: Potential for adaptive evolution at species range margins: contrasting interactions between red coral populations and their environment in a changing ocean
Source: Ecol Evol. 2015 Feb 20;5(6):1178–92. doi: 10.1002/ece3.1324 (PMC4377262; doi:10.1002/ece3.1324)
Supplement: Supplementary file 1 [file ece30005-1178-sd1.docx]

**Appendix S1: Experimental plates and calcein labeling**

Each colony was mounted in experimental plates. These plates were built up with three PVC disks (10 cm in diameter) and two rubber layers used to separate the disks. Each PVC disks had 8 holes (1 cm in diameter and one colony per hole). Plates and rubber layers were hold together with screws. The colonies were fixed randomly in the experimental plates without putty. They were hold mechanically thanks to the perpendicular cuts previously done in the rubber layers. All the plates were placed in a 10 litres incubator with one gram of calcein (Sigma C-0875) to allow quantification of the growth in diameter during the experimentation period. This protocol was realized using scuba diving in less than one hour.

After 24 hours in the incubator, the plates were then screwed onto a metallic bar (10 cm in height) fixed to the rock at their final depth following the experimental design. The two sets of plates corresponding to each treatment (i.e. control and transplant) were fixed in the same area (i.e. less than one meter square) to keep the colonies under the same environmental conditions.
