# Supplementary material for: Potential for adaptive evolution at species range margins: contrasting interactions between red coral populations and their environment in a changing ocean
Source: Ecol Evol. 2015 Feb 20;5(6):1178–92. doi: 10.1002/ece3.1324 (PMC4377262; doi:10.1002/ece3.1324)
Supplement: Supplementary file 2 [file ece30005-1178-sd2.docx]

**Appendix S2: Analysis of the growth in diameter**

Following Marschal *et al.* (2004) and Torrents (2007), the air-dried portion of the colony was embedded in epoxy resin (Durcupan ACM FLUKA) and heated to 60 °C for 24 hours. One millimeter thick cross sections were then sliced from each colony with a diamond saw. The sections were glued (ESCIL D200 SF UV radiation polymerization) to a microscope slide, and polished to obtain 150 μm thick slabs. We took photos of the section under epifluorescence microscopy (excitation filter 340-380nm) with a stereomicroscope (Leica © MZ16) connected to a video camera 6 (EvolutionTM LC Color – PLA662). The growth measurement were carried out with Visilog© 6.0 – TNPC© 4.1 using appropriate calibration. To avoid problem due to growth variability within colonies (Torrents 2007), we used stereomicroscope images obtained from different areas of each section and computed the mean of the 10 highest distances between the calcein label and the periphery of the section (mean maximum growth in the main text).

**REFERENCES:**

Marschal, C., Garrabou, J., Harmelin, JG. and Pichon, M. 2004. A new method for measuring growth and age in the precious red coral *Corallium rubrum* (L.). *Coral Reefs*. 23:423-432.

Torrents, O. 2007. Biologie des populations du corail rouge *Corallium rubrum* (L. 1758) de Méditerranée nord-occidentale. PhD Dissertation, Université de la Méditerranée, France.
