# Supplementary material for: Potential for adaptive evolution at species range margins: contrasting interactions between red coral populations and their environment in a changing ocean
Source: Ecol Evol. 2015 Feb 20;5(6):1178–92. doi: 10.1002/ece3.1324 (PMC4377262; doi:10.1002/ece3.1324)
Supplement: Supplementary file 3 [file ece30005-1178-sd3.docx]

**Appendix S3: Null allele frequencies, microsatellite characteristics, Hardy-Weinberg equilibrium and genetic diversity.**

The analysis conducted with MICROCHECKER v2.2.3 (Van Oosterhout *et al.* 2004) confirmed the absence of large allele dropout and scoring errors due stuttering in our dataset. We estimated the frequencies of null alleles (*r*) for each locus by the expectation maximization algorithm (Dempster *et al.* 1977) implemented in FREENA (Chapuis and Estoup 2007). Null allele frequencies ranged from 0 for *Mic26*, *Mic24* and *COR46bis* to 0.62 for *COR58*, with a mean value equal to 0.24 ± 0.21. To avoid bias due to null alleles, the genetic analyses were performed with seven loci (*Mic13*, *Mic20*, *Mic22*, *Mic24*, *Mic26*, *Mic27*, *COR46bis*), for which the mean *r* was < 0.1 (see Chapuis *et al.* 2008). Based on these seven microsatellites, we looked for the occurrence of null alleles in each sample using MICROCHECKER v2.2.3. Null alleles were present in all the samples except PZ-20.

Departure from panmixia was tested for each sample using the score test for heterozygote deficiency. Significance was addressed using a Markov-chain algorithm (Guo and Thompson 1992; Raymond and Rousset 1995) with default parameters. The *f* estimator of *FIS* (Weir and Cockerham 1984) was computed for each sample. Computations were done using GENEPOP v.4.0 (Rousset 2008). We tested the null hypothesis of linkage equilibrium for each pair of loci in each sample using the permutation procedure (*n* = 1000) implemented in GENETIX v.4.05 (Belkhir *et al.* 2004). Observed heterozygosity (*H_o_*) and gene diversity (*H_e_*; Nei 1973) were computed for each sample using GENETIX. We used ADZE (Szpiech *et al.* 2008) to compute the allelic richness (*Ar_(g)_*) for each sample with a rarefaction method (Petit *et al.* 1998) and *g*, the minimum number of genes at one locus in one of the samples, set to 64.

**REFERENCES:**

Belkhir, K., Borsa, P., Chikhi, L., Raufaste, N. and Bonhomme, F. 2004. GENETIX 4.05, logiciel sous Windows TM pour la génétique des populations. Laboratoire Génome, Populations, Interactions, CNRSUMR5000, Université de Montpellier II, Montpellier, France.

Chapuis, MP. and Estoup, A. 2007. Microsatellite null alleles and estimation of population differentiation. *Mol. Biol. Evol.* 24: 621-631.

Chapuis, MP., Lecoq, M., Michalakis, Y., Loiseau, A., Sword, GA., Piry, S., *et al.* 2008. Do outbreaks affect genetic population structure? A worldwide survey in *Locusta migratoria*, a pest plagued by microsatellite null alleles. *Mol. Ecol.* 17: 3640-3653.

Dempster, A., Laird, N. and Rubin, D. 1977. Maximum likelihood from incomplete data via the EM algorithm. *J. Roy. Stat. Soc. B*. 39:1-38.

Guo, S. and Thompson, E. 1992. Performing the exact test of Hardy-Weinberg proportion for multiple alleles. *Biometrics*. 48: 361-372.

Nei, M. 1973. Analysis of gene diversity in subdivided populations. *P. Nat. Acad. Sc. USA*. 70: 3321-3323.

Petit, RJ., Mousadik, AE. and Pons, O. 1998. Identifying populations for conservation on the basis of genetic markers. *Conserv. Biol.* 12: 844-855.

Raymond, M. and Rousset, F. 1995. GENEPOP ver. 1.2: a population genetics software for exact test and ecumenicism. *J. Hered.* 86: 248-249.

Rousset, F. 2008. GENEPOP’007: a complete re-implementation of the GENEPOP software for Windows and Linux. *Mol. Ecol. Resour.* 8: 103-106.

Szpiech, ZA., Jakobsson, M. and Rosenberg, NA. 2008. ADZE: a rarefaction approach for counting alleles private to combinations of populations. *Bioinformatics*. 27: 2498-2504.

Van Oosterhout C, Hutchinson WF, Wills DPM, Shipley P (2004) MICRO-CHECKER: software for identifying and correcting genotyping errors in microsatellite data. *Molecular Ecology Notes*. 6 : 255–256.

Weir, BS. and Cockerham, CC. 1984. Estimating F-statistics for the analysis of population structure. *Evolution*. 38: 1358-1370.
