# Supplementary material for: Potential for adaptive evolution at species range margins: contrasting interactions between red coral populations and their environment in a changing ocean
Source: Ecol Evol. 2015 Feb 20;5(6):1178–92. doi: 10.1002/ece3.1324 (PMC4377262; doi:10.1002/ece3.1324)
Supplement: Supplementary file 4 [file ece30005-1178-sd4.docx]

**Appendix S4: R-scripts for the PST-FST comparisons and corresponding sensitivity analyses.**

###Example for RTE conducted between RIO20 vs RIO40 : results obtained at 20 m###

# Computation MS obs:

dist.r <- seq(0,1,by=0.0001) #see expression for PST

growth <- c(0.1028, 0.12963, 0.06523, 0.08879, 0.09446, 0.08666, 0.10209, 0.08717, 0.13931, 0.11118, 0.09091, 0.09213, 0.04487, 0.06394, 0.08679, 0.07054, 0.05124, 0.0476, 0.05367, 0.05029); pop <- c("RIO20", "RIO20", "RIO20", "RIO20", "RIO20", "RIO20", "RIO20", "RIO20", "RIO20", "RIO20", "RIO40", "RIO40", "RIO40", "RIO40", "RIO40", "RIO40", "RIO40", "RIO40", "RIO40", "RIO40") #Dataset

require(nlme)

temp=lme(growth~1, random=~1|pop)

#PST for dist.r=1

varpop.obs <- as.numeric(VarCorr(temp)[1])

varres.obs <- as.numeric(VarCorr(temp)[2])

pst=(varpop.obs)/((varpop.obs)+(2*varres.obs))

#bootstrap PST-confidence interval:

#définition dataset

RIO20 <-c(0.1028, 0.12963, 0.06523, 0.08879, 0.09446, 0.08666, 0.10209, 0.08717, 0.13931, 0.11118); RIO40 <-c(0.09091, 0.09213, 0.04487, 0.06394, 0.08679, 0.07054, 0.05124, 0.0476, 0.05367, 0.05029)

nperm<-1000

growthboot<-matrix(NA,nrow=nperm,ncol=20)

MS.bootpop<-matrix(NA,nrow=nperm,ncol=1)

MS.bootres<-matrix(NA,nrow=nperm,ncol=1)

tablePSTboot<-matrix(NA,nrow=nperm,ncol=1)

#Définition IC 95: boucle

for(i in 1:nperm){

growthboot[i,] <- c((sample(RIO20,10, replace=T)), (sample(RIO40,10, replace=T)))

growthb <- growthboot[i,]

temp<- lme(growthb~1, random=~1|pop)

MS.bootpop[i,] <-as.numeric(VarCorr(temp)[1])

MS.bootres[i,] <-as.numeric(VarCorr(temp)[2])

tablePSTboot[i,]=(as.numeric(VarCorr(temp)[1])/(as.numeric(VarCorr(temp)[1])+2*as.numeric(VarCorr(temp)[2])))

}

#extraction MS.bootpop/res.min/max:

quantpop <- quantile(MS.bootpop, c(0.025,0.975))

MS.bootpop.min <-c(quantpop[[1]][1])

MS.bootpop.max <- c(quantpop[[2]][1])

quantres <- quantile(MS.bootres, c(0.025,0.975))

MS.bootres.min <-c(quantres[[1]][1])

MS.bootres.max <- c(quantres[[2]][1])

#plot PST function de r (=c/h2):

#PSTobs --> fonction r

PSTobs <- (dist.r*varpop.obs)/((dist.r*varpop.obs)+(2*varres.obs))

PSTobs.ord <- sort(PSTobs)

plot(dist.r,PSTobs.ord,type="l", lty="solid", lwd=3, col="black", xlab="r", ylab="PST/FST", main= "PST-FST comparison function of c/h2 for RIO20 vs. RIO40", xlim=0:1, ylim=0:1)

legend("topleft",lty=c("solid", "dotted", "dashed","dotted"), lwd=c(3,2,2,2), legend=c("PST", "95% CI", "Upper bound of 95% CI of FST", "FSTall2"), col=c("black","grey","black", "black"))

#PSTmin --> fonction r

PSTmin <- (dist.r* MS.bootpop.min)/(dist.r*MS.bootpop.min+2*MS.bootres.min)

PSTmin.ord <- sort(PSTmin)

lines(dist.r,PSTmin.ord,type="l", lty="dotted", lwd=2, col="grey")

#PSTmax --> fonction r

PSTmax <- (dist.r* MS.bootpop.max)/(dist.r*MS.bootpop.max+2*MS.bootres.max)

PSTmax.ord <- sort(PSTmax)

lines(dist.r,PSTmax.ord,type="l", lty="dotted", lwd=2, col="grey")

abline(h=0.03, lty="dashed", lwd=2, col="black")

abline(h=0.1, lty="dotted", lwd=2, col="black")
