# Supplementary material for: Potential for adaptive evolution at species range margins: contrasting interactions between red coral populations and their environment in a changing ocean
Source: Ecol Evol. 2015 Feb 20;5(6):1178–92. doi: 10.1002/ece3.1324 (PMC4377262; doi:10.1002/ece3.1324)
Supplement: Supplementary file 5 [file ece30005-1178-sd5.docx]

**Table S1**

a)

|  | Riou | | | Palazzu | |
| --- | --- | --- | --- | --- | --- |
|  | 5 m (2004-2006) | 20 m (2003- 2006) | 40 m (2000-2006) | 20m (2004-2007) | 40 m (2004-2007) |
| Mean T (°C) | 20.3±0.6 | 18.3±0.4 | 15.7±0.3 | 21.0±0.6 | 16.4±1.2 |
| Mean CV | 17.7±0.6 | 16.3±2.2 | 12.1±2.3 | 9.1±2.4 | 7.7±0.9 |
| Min | 13.7 | 13.5 | 13.0 | 14.2 | 13.2 |
| Max | 26.5 | 25.4 | 23.6 | 25.9 | 23.0 |
| % >20 | 60.7±4.7 | 33.9±8.6 | 5.6±4.1 | 68.1±17.1 | 3.1±4.4 |
| % >24 | 15.6±6.7 | 1.0±1.5 | 0 | 3.8±5.6 | 0 |

Table S1 a) Mean temperature conditions from July 1st to September 30th: mean, coefficient of variation (CV) and percentage of time with temperature higher or equal to 20 and 24 ºC at 5, 20 and 40 m depth at Riou and 20 and 40 m depth at Palazzu. Means were computed over the summer periods of 2004 to 2006 for Riou 5 m, 2003 to 2006 for Riou 20 m, 2000-2006 for Riou 40m and 2004-2007 for both depths at Palazzu. This table was constructed based on the analysis of high-resolution temperature time series published in Bensoussan *et al.* (2010).

|  | Riou 5 m (2006) |
| --- | --- |
|  |  |
| Mean T (°C) | 20.9±3.6 |
| Min | 13.7 |
| Max | 28.6 |
| % >20 | 65.7 |
| % >24 | 19.8 |

Table S1 b) Mean and maximum temperature values and percentage of time with temperature higher or equal to 20 and 24°C at 5 m depth for the 2006 summer at Riou. The temperatures of 24 °C were proposed by Torrents *et al.* (2008) as upper thermal threshold for *Corallium rubrum*. This table was constructed based on the analysis of high-resolution temperature time series published in Bensoussan *et al.* (2010).

**REFERENCES:**

Bensoussan, N., Romano, JC., Harmelin, JG. and Garrabou, J. 2010. High resolution characterization of northwest Mediterranean coastal waters thermal regimes: to better understand responses of benthic communities to climate change. *Estuar. Coast. Shelf S.* 87: 431-441.

Torrents, O., Tambutté, E., Caminiti, N. and Garrabou, J. 2008. Upper thermal thresholds of shallow vs. deep populations of the precious Mediterranean red coral *Corallium rubrum* (L.): Assessing the potential effects of warming in the NW Mediterranean. *J. Exp. Mar. Biol. Ecol.* 357: 7-19.
