# Supplementary material for: Potential for adaptive evolution at species range margins: contrasting interactions between red coral populations and their environment in a changing ocean
Source: Ecol Evol. 2015 Feb 20;5(6):1178–92. doi: 10.1002/ece3.1324 (PMC4377262; doi:10.1002/ece3.1324)
Supplement: Supplementary file 6 [file ece30005-1178-sd6.docx]

**Table S2**

|  | RI-20 | RI-40 | PZ-20 | PZ-40 |
| --- | --- | --- | --- | --- |
| RI-20 |  |  |  |  |
| RI-40 | **0.02 (0.01 - 0.03)** |  |  |  |
| PZ-20 | **0.14 (0.09 - 0.19)** | **0.12 (0.08 - 0.17)** |  |  |
| PZ-40 | **0.12 (0.08 - 0.16)** | **0.11 (0.06 - 0.16)** | **0.1 (0.05 - 0.16)** |  |

Table S2: Pairwise *θ* values and corresponding 95% CI interval are shown below the diagonal. All values are significantly different from 0.
